# Health information sources for different types of information used by Chinese patients with cancer and their family caregivers

**DOI:** 10.1111/hex.12498

**Published:** 2016-09-07

**Authors:** Bo Xie, Zhaohui Su, Yihao Liu, Mo Wang, Ming Zhang

**Affiliations:** ^1^ School of Nursing & School of Information The University of Texas at Austin Austin TX USA; ^2^ Department of Advertising and Public Relations The University of Texas at Austin Austin TX USA; ^3^ Department of Management Warrington College of Business Administration University of Florida College Park MD USA; ^4^ Department of Oncology Sichuan Provincial People's Hospital Sichuan China

**Keywords:** cancer patient care, health information sources, internet use, patient participation, patient‐centred care

## Abstract

**Context:**

Little is known about the information sources of Chinese patients with cancer and their family caregivers, yet this knowledge is critical for providing patient‐centred care.

**Objective:**

To assess and compare the information sources used by Chinese patients with cancer and their family caregivers.

**Design:**

The validated Health Information Wants Questionnaire (HIWQ) was translated and administered in March 2014.

**Setting:**

The oncology department of a general hospital in south‐west China.

**Participants:**

A convenience sample of 198 individuals, including 79 patients with cancer (mean age=55.24, SD=13.80) and 119 family caregivers (mean age=46.83, SD=14.61).

**Main outcome measures:**

Ratings on the HIWQ items assessing information sources for different types of information.

**Results:**

The interaction between information source and group was significant (*F*_3,576_=6.32, *P*<.01). Caregivers obtained more information than patients from the Internet. Caregivers and patients did not differ in the amount of information they obtained from doctors/nurses, interpersonal contacts or mass media. The interaction between information type and information source was significant (*F*_18,3456_=6.38, *P*<.01). Participants obtained more information of all types from doctors/nurses than from the other three sources and obtained more information from interpersonal contacts than from mass media or the Internet.

**Conclusions:**

The information sources of Chinese patients with cancer and their family caregivers were similar, with an important difference that caregivers obtained more online information than patients. These findings have important implications for patient care and education in China where the family typically plays a major role in the care and decision making.

## 1 Introduction

Patient‐centred care requires an understanding of the sources patients and their family caregivers use to obtain different types of health information. Such knowledge can inform health‐care professionals and educators to develop effective interventions and strategies to help patients and their family caregivers obtain high‐quality health information and participate in health‐care decisions about themselves and their loved ones.

Cancer has become a top killer in China, with over three million Chinese people being newly diagnosed with cancer and nearly two million die of cancer annually.^1^ Yet little is known about the health information sources Chinese patients with cancer and their family caregivers use to obtain different types of information. Research to date has focused primarily on the health information behaviours of cancer patients while understudying those of family caregivers,^2^ despite the fact that cancer caregivers often carry heavy burdens of care and are in great need of various types of information to help themselves and their loved ones cope with and manage the condition.^3^, ^4^, ^5^, ^6^

The limited evidence on cancer caregivers suggests that caregivers’ health information behaviours are similar to those of patients with cancer. These include that, first, medical professionals are the primary information source for patients with cancer and caregivers;^2^, ^7^, ^8^, ^9^, ^10^ second, patients with cancer and caregivers typically are unable to obtain sufficient information from their health‐care providers;^11^, ^12^, ^13^, ^14^, ^15^ and third, other information sources (e.g. interpersonal contacts with friends and family, mass media, and the Internet) are commonly used by patients with cancer and caregivers to help meet their many unmet information needs.^2^, ^7^, ^8^, ^9^, ^10^, ^16^, ^17^

In terms of the types of health information wanted and sought by patients with cancer and caregivers, existing research tends to predominately examine a limited range of types of information, particularly those about the diagnosis/status of the illness and treatment plans and options, while understudying other types of information that are also important to patients and caregivers, for example information about how to cope with cancer psychologically and socially.^8^, ^11^, ^12^, ^14^, ^15^ Our prior work on Health Information Wants (HIW) represents an important exception to this predominant approach. HIW is defined as ‘health information that one would like to have and use to make important health decisions that may or may not be directly related to diagnosis or standard treatment’^18^ (p. 5.14). This concept was developed with the intention to cover a broad range of types of health information that patients typically would be interested in health‐care contexts. This broad coverage differentiates the HIW concept from the previously predominant perspective that health‐care providers typically have: from the provider's perspective, patients *need* to have only a limited range of information (which typically focus on compliance).^18^, ^19^, ^20^ From the patient's perspective, however, there is often a wide range of types of information that patients *want* to have for reasons that may, or may not, be related to compliance, for example information about coping with cancer. The HIW concept therefore promotes an understanding of patient preferences from the perspective of the patient instead of the provider.

Our subsequently developed HIW Questionnaire (HIWQ) operationalizes patients’ desire for health information to seven specific aspects of medical encounters: diagnosis, treatment, laboratory testing, self‐care, complementary and alternative medicine (CAM), psychosocial aspects and health‐care providers. The validity and reliability of the HIWQ are supported by empirical evidence from college students and community‐dwelling older adults in the United States.^21^, ^22^

Our previous HIWQ‐related work focused on patients only and did not take into account family caregivers. This study represents our first effort to translate and adapt the HIWQ to the Chinese context. In particular, in recognition of the important role Chinese families typically play in cancer patient's information seeking, decision making and coping,^23^, ^24^, ^25^ we expanded the original HIWQ to also assess Chinese family caregivers’ information wants and behaviours. The first paper derived from this study, focusing on comparing differences in types of information patients and family caregivers *wanted* vs *obtained from medical professionals*, was reported elsewhere.^26^ The present paper focuses instead on assessing and comparing the health information sources used by Chinese patients with cancer and their family caregivers for different types of information. Advancing this knowledge is important because it might inform clinical practice and health education interventions, leading to improved patient care quality and outcomes (e.g. if evidence suggested patients tended to obtain one particular type of information from one particular source while family caregivers tended to obtain the same type of information from a different source, then health‐care professionals might want to develop interventions targeting the different sources to ensure patients and their families obtaining the necessary information). The primary research questions guided the present paper were as follows:

- What are the information sources used by Chinese patients with cancer and their family caregivers for different types of health information and what differences and/or similarities might there be between these two groups in the information sources they use for different types of health information?

## 2 Method

### 2.1 Research site

Participants were recruited from the Oncology Department of the Sichuan Academy of Medical Sciences and Sichuan Provincial People's Hospital (referred to as the ‘Hospital’ thereafter). The Hospital is a top‐rated large general hospital located in Chengdu, Sichuan, China. It has with over 3000 beds serving patients primarily from south‐west China, an economically less developed region. Its Oncology Department has 124 beds for patients diagnosed with a variety of cancer types and stages.

### 2.2 Design and materials

This study was a cross‐sectional, paper‐based survey study of Chinese patients with cancer and their family caregivers. The validated 21‐item HIWQ,^21^, ^22^ developed based on the HIW concept as reviewed above,^18^ was translated and adapted for this study. The HIWQ includes two parallel scales, with one measuring preferences for different types of information (the Information Preference Scale/IPS) and the other measuring preferences for participation in the corresponding types of decision making (the Decision‐making Preference Scale). Due to the specific scope of the present study, we adapted only the IPS in this study. The IPS contains seven subscales with items in each subscale measuring one specific type of health information with a 5‐point Likert‐type scale ranging from 1=None to 5=All. These include information about: diagnosis (items 1–4), treatment (items 5–7), laboratory testing (items 8–10), self‐care (items 11–13), CAM (items 14–16), psychosocial aspects (items 17–19) and health‐care providers (items 20–21). The original English version of the 21‐item HIWQ is freely available online.^21^

A bilingual translator translated these original HIWQ items into Chinese, which was then verified and revised by the first author for accuracy and consistency. To answer the research questions of this study, the following new survey questions were added to each of the original 21 items: How much of this type of the information have you already obtained from (i) a doctor/nurse; (ii) a family/friend/neighbour/acquaintance; (iii) newspaper/magazine/television/radio; and (iv) the Internet? The format of the instrument was also revised to accommodate these new questions. Table 1 below illustrates a sample item with the original formatting from the original English instrument, while Table 2 illustrates the same sample item from the revised Chinese instrument.

**Table 1:** A sample item from the original Health Information Wants Questionnaire

How much information would you like to have?					
Information about how severe this health condition is	None	A little	Some	Most	All

**Table 2:** The corresponding sample item from the Chinese instrument

Information about how severe this health condition is					
a. How much of this type of information would you like to have?	None	A little	Some	Most	All
b. How much of this type of the information have you already obtained from a doctor/nurse?	None	A little	Some	Most	All
c. How much of this type of the information have you already obtained from a family/friend/neighbour/acquaintance?	None	A little	Some	Most	All
d.How much of this type of the information have you already obtained from newspaper/magazine/television/radio?	None	A little	Some	Most	All
e.How much of this type of the information have you already obtained from the Internet?	None	A little	Some	Most	All

To determine any problematic items of the Chinese instrument, we conducted multiple rounds of testing using the cognitive interviewing technique, which is commonly used in fields such as health sciences and psychology to validate questionnaire items among intended populations.^27^, ^28^ Our cognitive interviewing was done with, first, a total of nine graduate students and visiting scholars that were all native Chinese speakers studying in the United States. Next, we conducted cognitive interviewing with two older Chinese women living in Beijing, followed by testing with three medical students interning at the Hospital. Finally, we conducted two rounds of cognitive interviewing with seven participants from the Hospital (four patients with cancer and three family caregivers; data from these participants were excluded from our final data set). We revised the instrument based on the results from each round of the cognitive interviewing. This Chinese instrument has excellent validity and reliability, as reported in detail in our first paper from this study.^26^ A full copy of the questionnaire used in this study can be obtained upon request to the first author.

### 2.3 Participants

Participants were recruited from patients of our research site, and family caregivers accompanying these patients during the time of data collection, March 2014. Inclusion criteria included the following: (i) at least 18 years of age; (ii) able to read, write and speak Chinese; and (iii) normal hearing and vision, corrected or uncorrected. One of the authors (MZ), an oncology physician with 20 years of experience working at the Hospital, recruited participants through word‐of‐mouth. A total of 79 patients with cancer and 119 caregivers completed the instrument (*N*=198; some patients were accompanied by more than one family caregiver; we allowed multiple caregivers of a patient to participate if they all consented to).

### 2.4 Procedure

This study was approved by the University of Texas at Austin Institutional Review Board. Informed consent was obtained prior to any data collection. Each participant completed a hardcopy questionnaire independently. The average time for completing the instrument was approximately 20–25 minutes for patients and 15–20 minutes for caregivers. Upon completion, we thanked each participant and gave each a small hand towel as our token of appreciation.

### 2.5 Data analysis

Three‐way mixed ANOVA was conducted in the SPSS 22.0 software, (IBM Corp., Armonk, NY, USA), with one between‐subject factor (group: patents vs caregivers) and two within‐subject factors (type of information; source of information). Consistent with our previous research,^22^ participants’ original responses to all HIWQ items were rescaled to range from 0 (corresponding to the least amount of information obtained) to 100 (corresponding to the most amount of information obtained) with a mid‐point of 50. The formula used for the rescaling was as follows: rescaled score=(raw score−1)*25. Then, we averaged the rescaled items measuring the same type of health information and created a scale score for each type of health information obtained from each source (i.e. 28 scale scores were created for each participant, representing the seven types of health information obtained from each of the four sources of study).

Additionally, to further examine the relationship between general Internet use and the amount of information patients and caregivers obtained from different sources, we conducted two sets of one‐way ANOVA. In the first ANOVA, the grouping factor was Internet use history (recoded as 1=‘Never’ or ‘Less than a year’, 2=‘1–3 years’ or ‘3–5 years’, 3=‘5–10 years’ or ‘more than 10 years’ to make the number of responses in each cell more balanced). In the second ANOVA, the grouping factor was Internet use frequency (recoded as 1=‘Never’, 2=‘Less than once per month’, ‘More than once per month’, ‘Once per month’, or ‘Once per 2–3 days’, and 3=‘Everyday’). The dependent variables were information obtained averaged across all seven types from each of the four sources.

## 3 Results

Patients’ age range was 22–82 years (m*ean*=55.24, m*edian*=59.00, 25% percentile=46.00, 75% percentile=65.00, *SD*=13.80) and caregivers’ was 21–78 (m*ean*=46.83, m*edian*=44.00, 25% percentile=35.00, 75% percentile=59.00, *SD*=14.61). Women accounted for 42% of the patient and 53% of the caregiver groups. The majority of patients and caregivers were married (82% and 88%, respectively). The majority of patients (75%) reported their overall health status being Okay or Good, and 10% reported having Very Good or Extremely Good health status. In comparison, a quarter of the caregivers reported their overall health status being Very Good or Extremely Good. The majority of caregivers (65%) had at least high school education, while the majority of patients (54%) had below high school education. Almost all patients (98%) reported having very low (15%), low (42%) or medium (41%) household income compared with other families in the region. Similarly, 92% of family caregivers reported having very low (6%), low, (35%) or medium (51%) household income. The majority of patients (82%) and caregivers (77%) had health insurance coverage. The top three cancer types patients were diagnosed with were breast cancer (23%), lung cancer (22%), and colon and rectal cancer (11%). The mean and median time patients had been diagnosed with cancer were 15.34 months and 6 months (25% Percentiles=4 month, 75% Percentile=12 months, SD=26.18). Participants’ Internet experience (i.e. Internet use history, Internet use frequency) is summarized in Table 3 above.

**Table 3:** Internet experience of study participants

Variable		Patient n=79	Caregiver n=119	Independent *t* test*
Internet use history: ‘How long has been using the Internet’				
	Never	44(57.9)**	37(32.2)	*t*=−4.25, *P*<.01
	<1 y	8(10.5)	5(4.3)	
	>1 y, <3 y	3(3.9)	7(6.1)	
	>=3 y, <5 y	6(7.9)	16(13.9)	
	>=5 y, <10 y	7(9.2)	23(20.0)	
	>10 y	7(9.2)	25(21.7)	
	Other	1(1.3)	2(1.7)	
Internet use frequency: ‘How often uses the Internet’				
	Never	43(57.3)	35(31.0)	*t*=−4.57, *P*<.01
	Less than once a month	6(8.0)	2(1.8)	
	At least once a month	1(1.3)	3(2.7)	
	Once a week	7(9.3)	11(9.7)	
	Every 2–3 days	4(5.3)	14(12.4)	
	Every day	14(18.7)	48(42.5)	

*A positive *t*‐value represented that the patients’ value was higher than that of the caregivers, while a negative *t*‐value represented that the patients’ value was lower than that of the caregivers.

**The first number in each cell is the number of participants; percentages are in parentheses.

Difference tests between the two groups (independent *t* tests for interval variables and chi‐square tests for categorical and ordinal variables) show that, compared with the caregivers, patients were significantly older, had lower overall health status, less education, lower household income, shorter Internet use history and lower Internet use frequency. These two groups did not differ significantly in other aspects of their demographic characteristics. Specific details about these difference tests are reported in the first paper derived from this project.^26^ The amount of the seven types of information obtained by patients and by caregivers from the four sources is reported in Table 4 below.

**Table 4:** Amount of the seven types of information obtained by patients and caregivers from different sources

Subscale	Patients				Caregivers			
	Doctors/nurses	Family/neighbour/acquaintance	Newspaper/magazine/television/radio	Internet	Doctors/nurses	Family/neighbour/acquaintance	Newspaper/magazine/television/radio	Internet
Diagnosis	47.65(22.20)	36.23(24.87)	33.01(25.44)	23.18(25.76)	51.91(22.58)	37.71(25.59)	33.97(25.59)	34.68(29.88)
Treatment	44.46(24.36)	27.99(24.54)	25.32(25.95)	20.15(26.51)	46.22(27.12)	30.54(26.55)	27.93(28.24)	29.48(29.61)
Laboratory tests	49.05(25.60)	29.59(26.98)	23.00(28.35)	16.77(25.99)	49.72(28.68)	31.82(28.08)	28.81(29.51)	31.21(30.99)
Self‐care	50.21(25.29)	36.34(24.78)	27.37(26.16)	19.83(27.46)	50.14(27.20)	37.01(26.10)	32.51(28.35)	31.86(30.31)
CAM	42.83(58.41)	29.11(25.59)	23.84(27.50)	17.51(27.43)	38.17(30.39)	30.01(26.65)	26.78(27.76)	25.78(29.17)
Psychosocial factor	41.56(24.62)	31.80(23.94)	25.79(25.56)	18.51(26.19)	43.00(28.50)	31.99(26.31)	28.70(27.17)	26.98(28.68)
Health‐care provider	46.68(26.23)	37.82(24.76)	26.11(26.64)	16.67(24.73)	49.26(30.20)	40.36(28.42)	33.01(31.42)	30.08(30.89)

The first number in each cell is the mean amount; standard deviations are in parentheses. The original scores were rescaled with 0 representing the least amount of information obtained and 100 representing the most amount of information obtained; see the Data Analysis section above. CAM: Complementary and Alternative Medicine.

Our three‐way ANOVA found a significant interaction between source of information and group (*F*_3,576_=6.32, *P*<.01). Caregivers obtained more information from the Internet than did patients (diff=10.73, *P*<.01). These two groups had no statistically significant difference in the amount of information they obtained from doctors/nurses (diff=.70, *P*=.84), interpersonal contacts (diff=1.25, *P*=.71) or mass media (diff=3.55, *P*=.33). Thus, this significant interaction was driven by the difference in information obtained from the Internet between caregivers and patients.

The interaction between type of information and source of information was also significant (*F*_18,3456_=6.38, *P*<.01). Post hoc analyses showed that participants obtained more information of all seven types from doctors/nurses than from each of the other three sources and obtained more information from interpersonal contacts than from mass media or the Internet. The amount of treatment information and laboratory testing information participants obtained from mass media did not differ from that obtained from the Internet (diff=1.21, *P*=.40, and diff=1.06, *P*=.50, respectively). However, participants obtained more information of the other five types from mass media than from the Internet.

The three‐way interaction between group, source of information and type of information was not significant (*F*_18,3456_=1.41, *P*=.12). Neither was the interaction between type of information and group (*F*_6,1152_=.847, *P*=.542).

Additionally, the main effect of type of information was significant (*F*_6,1152_=10.78, *P*<.01). Pair comparison analyses showed that, overall, participants obtained more information about diagnosis and health‐care providers than that about treatment (diff=5.68, *P*<.01 for diagnosis information; diff=3.42, *P*<.01 for health‐care provider information), laboratory testing (diff=4.76, *P*<.01 for diagnosis information; diff=2.49, *P*<.05 for health‐care provider information), self‐care information (diff=5.04, *P*<.01 for diagnosis information; diff=2.78, *P*<.05 for health‐care provider information), CAM (diff=8.05, *P*<.01 for diagnosis information; diff=5.79, *P*<.01 for health‐care provider information) and psychosocial aspects (diff=6.50, *P*<.01 for diagnosis information; diff=4.24, *P*<.01 for health‐care provider information). In addition, participants obtained more information about laboratory testing (diff=3.29, *P*<.01) and self‐care (diff=3.01, *P*<.05) than that about CAM. No other pair comparison analyses were significant. In short, overall, participants had obtained more information about diagnosis and health‐care providers than that about the other five types of information.

The main effect of source of information was significant (*F*_3,576_=94.13, *P*<.01). Among the four sources, medical professionals provided more information than interpersonal contacts (diff=12.63, *P*<.01), mass media (diff=17.63, *P*<.01) and the Internet (diff=21.38, *P*<.01); interpersonal contacts provided more information than mass media (diff=4.99, *P*<.01) and the Internet (diff=8.75, *P*<.01). Mass media provided more information than the Internet (diff=3.75, *P*<.01). Therefore, medical professionals were the most important information source for our participants, followed by interpersonal contacts, then mass media, and then the Internet. The main effect of group was not significant (*F*_1,192_=2.09, *P*=.15). Therefore, patients and their family caregivers did not differ in the total amount of information they obtained (Table 5)

**Table 5:** Summary of the three‐way ANOVA results

	*F*‐value/D	*P*‐value	Effect size
Major effects			
Type	*F*_6,1152_=10.78	<.01	0.05
Source	*F*_3,576_=94.13	<.01	0.33
Group	*F*_1,192_=2.09	.15	0.01
Type*Source	*F*_18,3456_=6.38	<.01	0.03
Type*Group	*F*_6,1152_=0.87	.52	0.01
Source*Group	*F*_3,576_=6.32	<.01	0.03
Type*Source*Group	*F*_18,3456_=1.41	.12	0.01
Pair comparisons of source			
Medical professionals—Interpersonal contacts	D=12.63	<.01	
Medical professionals—Mass media	D=17.63	<.01	
Medical professionals—Internet	D=21.38	<.01	
Interpersonal contacts—Mass media	D=4.99	<.01	
Interpersonal contacts—Internet	D=8.75	<.01	
Mass media—Internet	D=3.75	<.01	
Pair comparisons of type			
Diagnosis—Treatment	D=5.68	<.01	
Diagnosis—Laboratory test	D=4.76	<.01	
Diagnosis—Self‐care	D=5.04	<.01	
Diagnosis—CAM	D=8.05	<.01	
Diagnosis—Psychosocial factor	D=6.50	<.01	
Diagnosis—Health‐care provider	D=2.27	.09	
Treatment—Laboratory test	D=−0.92	.32	
Treatment—Self‐care	D=−0.64	.57	
Treatment—CAM	D=2.37	.06	
Treatment—Psychosocial factor	D=0.82	.41	
Treatment—Health‐care provider	D=−3.42	<.01	
Laboratory test—Self‐care	D=0.28	.78	
Laboratory test—CAM	D=3.29	<.01	
Laboratory test—Psychosocial factor	D=1.75	.09	
Laboratory test—Health‐care provider	D=−2.49	<.05	
Self‐care—CAM	D=3.01	<.05	
Self‐care—Psychosocial factor	D=1.46	.12	
Self‐care—Health‐care provider	D=−2.78	<.05	
CAM—Psychosocial factor	D=−1.56	.14	
CAM—Health‐care provider	D=−5.79	<.01	
Psychosocial factor—Health‐care provider	D=−4.24	<.01	

D, mean difference across conditions; Type, type of health information; Source, source of information obtained from; Group, patient vs caregiver.

The effect size index reported is partial eta squared.

**Figure 1:**
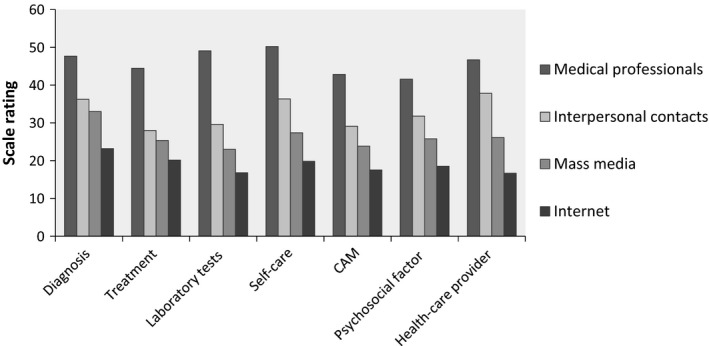
Information patients obtained from different sources

**Figure 2:**
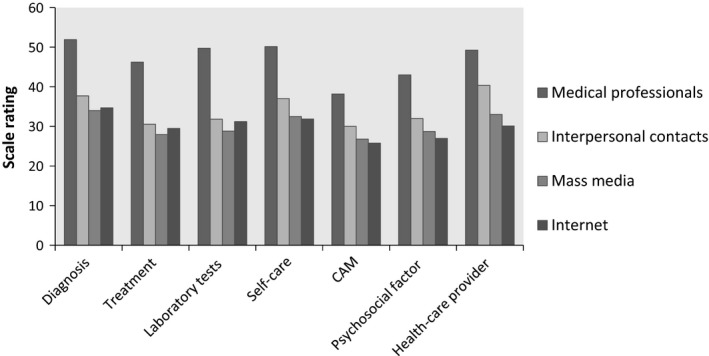
Information caregivers obtained from different sources

These findings are illustrated below in Fig. 1 (information obtained by patients) and Fig. 2 (information obtained by caregivers). The two sets of one‐way ANOVA on the relationship between general Internet use and the amount of information obtained from different sources found that the amount of information participants obtained from all four sources differed significantly at different conditions of Internet use history and Internet use frequency (Table 6).

**Table 6:** Internet use and information obtained from different sources

Dependent variables	Internet use history as the grouping factor	Internet use frequency as the grouping factor
Information obtained from medical professionals	*F*_2,185_=4.22*	*F*_2,185_=3.32*
Pair comparisons of the grouping factor		
1–2	D=−13.81**	D=−10.51*
1–3	D=−5.23	D=−6.77
2–3	D=8.58	D=3.74
Information obtained from interpersonal contacts	*F*_2,184_=9.86**	*F*_2,184_=7.94**
Pair comparisons of the grouping factor		
1–2	D=−18.20**	D=−12.18**
1–3	D=−11.21**	D=−13.76**
2–3	D=6.99	D=−1.58
Information obtained from mass media	*F*_2,184_=15.16**	*F*_2,184_=16.42**
Pair comparisons of the grouping factor		
1–2	D=−22.08**	D=−18.62**
1–3	D=−16.58**	D=−20.50**
2–3	D=5.50	D=−1.88
Information obtained from the Internet	*F*_2,185_=53.63**	*F*_2,185_=59.79**
Pair comparisons of the grouping factor		
1–2	D=−31.31**	D=−31.08**
1–3	D=−32.89**	D=−36.15**
2–3	D=−1.58	D=−5.07

‘D’=mean difference across conditions; For Internet use history, 1=‘Never’ or ‘Less than a year’, 2=‘1–3 y’ or ‘3–5 y’, and 3=‘5–10 y’ or ‘more than 10 y’; For Internet use frequency, 1=‘Never’, 2=‘Less than once per month’, ‘More than once per month’, ‘Once per month’, or ‘Once per 2–3 days’, and 3=‘Everyday’.

*P<.05, **P<.01.

Pair comparison tests showed that the significant effects were mainly driven by using the Internet or not, as indicated by the significant differences between Category 1 and Category 2 across all four sources and between Category 1 and Category 3 across some of the four sources for both Internet use variables. The differences between Category 2 and Category 3 were not significant across all four sources for neither of the two Internet use variables, suggesting that once participants started using the Internet, the amount of time spent on the Internet and the frequency of Internet use might not be related to the amount of health information they obtained.

## 4 Discussion

Our findings show that Chinese patients with cancer and their family caregivers obtained information from all four different sources, that is, medical professionals, interpersonal contacts, mass media and the Internet. Overall, participants obtained more information of all seven types, that is, diagnosis, treatment, laboratory testing, self‐care, complementary and alternative medicine (CAM), psychosocial aspects and health‐care providers, from medical professionals than from any of the other three sources, that is, interpersonal contacts, mass media or the Internet. These findings are in line with those reported in Western contexts where patients and caregivers use a variety of sources for health information,^2^, ^7^, ^8^, ^9^, ^10^, ^16^, ^17^ and medical professionals have consistently been reported as the most prominent source of information,^8^ even among those who also seek health information online.^9^, ^29^

Recent evidence suggests that in developed countries such as the United States the Internet has become the second most prominent source of information (after medical professionals, and more prominent than mass media or interpersonal contacts).^7^, ^9^ However, among our Chinese participants, the Internet was the least used source of information. Plausible reasons include, first, compared with the vast amount of high‐quality online information available to English speakers, much less such information is available in Chinese.^30^, ^31^, ^32^ Second, compared with their Western counterparts, Chinese participants may have lower levels of computer/Internet access and literacy.^33^ In fact, over 90% of our participants reported having very low‐to‐medium household income, and more than half of the patients and roughly one‐third of the caregivers had never used the Internet. Thus, our Chinese participants might not have had sufficient financial resources to secure access to computers and the Internet. An important implication of this finding is that future interventions aiming to provide Chinese patients with cancer and their families with health information may want to minimize their reliance on Internet connectivity. Rather, mobile phones used by 93% of the Chinese population 34 may be a better option in reaching a broader audience. (Note though, only 38% of the Chinese population had Internet access via smart phones.^35^ Mobile phone short message service, which does not rely on Internet connectivity, may be the best way to reach the most Chinese patients and caregivers, particularly those who cannot afford the more expensive smart phones.)

A novel contribution of this study was that, unlike previous studies that examined only a limited range of types of information, we explored a broad range of different types of information. Our results showed a significant interaction between type of information and source of information. While the amount of treatment and laboratory testing information participants obtained from mass media did not differ from that obtained from the Internet, they obtained more information of the other five types from mass media than from the Internet. A likely reason is that Chinese websites currently have more online information about cancer treatment and laboratory tests than the other five types of information. Future interventions should provide a broader range of types of information to help meet the diverse needs of Chinese patients with cancer and their families.

Our findings also suggest that Chinese patients with cancer and their family caregivers differed in their use of one of the health information sources: the Internet, with caregivers obtaining more information from the Internet than did patients (these two groups did *not* differ in the amount of information they were able to obtain from doctors/nurses, interpersonal contacts, or mass media). This finding is not surprising given that, compared with their caregivers, the patients in this study were significantly older and had lower levels of overall health condition, education, household income, and Internet use history and frequency, with these factors being shown to be negatively correlated to use of the Internet for health information.^16^ This finding is also in line with those reported in the literature,^36^ supporting its generalizability across populations and settings.

Our findings have important implications for health research and practice. Being able to obtain desired health information is essential to both patients with cancer and their family caregivers, as information can help reduce their distress (e.g. anxiety, depression) and cope with cancer‐associated challenges and uncertainties patients and their families typically encounter in their daily lives. This may help patients and their families have a less negative experience with the illness compared with situations where they have insufficient information.^14^, ^37^, ^38^, ^39^, ^40^ Our findings on the types of information Chinese patients with cancer and their family caregivers obtained from different sources can inform the design and development of interventions aiming at providing sufficient and desired information to patients and their families. For instance, if China follows the trends in Western countries, then the Internet would likely increasingly become a more prominent source of information for patients and their families. However, as our results suggest, currently Chinese patients and their families obtained less information about diagnosis, self‐care, CAM, psychosocial aspects and health‐care providers from the Internet than from mass media (the amount of treatment and laboratory testing information participants obtained from mass media did not differ from that obtained from the Internet). It would be important for any Internet‐based interventions to provide information about these currently underrepresented types of information so that patients and their families could obtain a broader range of the health information they wanted to make informed decisions.

Compared with the amount of research on patients with cancer, relatively little is known about cancer caregivers’ health information behaviours. Even less has examined potential differences between these two groups. Yet, as a recent review article points out, patients and caregivers mutually affect each other's emotional distress throughout the entire course of the illness.^41^ It is thus essential to consider both parties in any intervention that aims to improve the psychological well‐being and clinical outcomes of patients with cancer. By comparing the health information behaviours of patients with cancer and their caregivers, our findings can shed light on the design of interventions that can be effective to both patients and caregivers.

A major study limitation is that, using a convenience sample of patients and family caregivers from one Chinese hospital, the study findings’ generalizability is limited. In particular, the vast majority of our participants reported having very low‐to‐medium household income, and more than half of the patients and roughly one‐third of the caregivers had never used the Internet. These characteristics of our study sample are not surprising given that the main region the Hospital serves, that is, south‐west China, is economically less developed compared with those along the east coast of China. Future research should verify these findings in different Chinese patient with cancer and caregiver populations in different regions using, ideally, nationally representative samples of Chinese patients with cancer and caregivers. Still, the findings of our study may hold in Chinese populations that have, for instance, similar economic status and Internet experience. Also, this study used a cross‐sectional design, which provides only a snapshot view of patients’ and their family caregivers’ health information behaviours. Future research should explore whether and how these behaviours may evolve during the entire course of cancer management. To do so, longitudinal research would be necessary.

## 5 Conclusions

Our findings suggest that the health information sources of Chinese patients with cancer and their family caregivers were similar, with an important difference that, compared with patients, caregivers obtained more health information from the Internet. Still, for both the patients and caregivers groups, the Internet was the least used source of information compared with the other three sources of information. Importantly, while the amount of treatment and laboratory testing information participants obtained from mass media did not differ from that obtained from the Internet, they obtained more information of the other five types from mass media than from the Internet. These findings have important implications for patient care and education in China particularly as Chinese families typically play a major role in the care and decision making.

## Source of funding

The first author's faculty start‐up research funds from the University of Texas at Austin and her Ed and Molly Smith Centennial Fellowship in Nursing funded this study's data collection and data entry.

## Conflict of interests

The authors declare no conflict of interests.
